# Investigation of *HOXA9* promoter methylation as a biomarker to distinguish oral cancer patients at low risk of neck metastasis

**DOI:** 10.1186/1471-2407-14-353

**Published:** 2014-05-21

**Authors:** Kenichiro Uchida, Ratna Veeramachaneni, Bing Huey, Aditi Bhattacharya, Brian L Schmidt, Donna G Albertson

**Affiliations:** 1 Helen Diller Family Comprehensive Cancer Center, University of California San Francisco, 1450 Third Street, San Francisco CA 94158-9001, USA; 2 Bluestone Center for Clinical Research, New York University College of Dentistry, 421 First Avenue, New York NY 10010-4086, USA; 3 Current address, Department of Oral and Maxillofacial Surgery, Yamaghuchi University Hospital, 1-1-1,Minamikogushi, Ube City, Yamaguchi Prefecture 755-8505, Japan; 4 Current address, Department of Diagnostic Sciences Texas A&M HSC Baylor College of Dentistry, 3302 Gaston Ave, Room 214, Dallas, TX 75246, USA

**Keywords:** Oral cancer, Metastasis, *HOXA9*, Methylation, Pyrosequencing

## Abstract

**Background:**

Metastasis to the cervical (neck) lymph nodes is one of the most significant clinical factors responsible for death from oral squamous cell carcinoma (SCC). Therefore, the lymph nodes are frequently removed when the tumor is excised (neck dissection), even though the majority of patients will not benefit from the extra surgery. Two subtypes of oral SCC distinguished by the presence of tumor genomic aberrations +3q, -8p, +8q and/or +20 differ in risk for metastasis – high for the 3q8pq20 subtype, harboring one or more of the aberrations and low for the non-3q8pq20 subtype, lacking these alterations. A prior analysis of the literature suggested genes differentially methylated in the two subtypes. Therefore, the goal of this study was to further investigate the methylation status of candidate biomarkers of the non-3q8pq20 subtype, and evaluate their utility for identifying patients at low risk for metastasis.

**Methods:**

Methylation status of genes in a cohort of 52 oral SCC patients with at least five year follow up was determined by pyrosequencing. Gene expression levels were determined by quantitative RT-PCR. Growth following re-expression of *HOXA9* in cultured oral SCC cells was assessed by proliferation and colony formation assays.

**Results:**

A pilot study evaluating methylation levels of *HOXA9*, *MT1A* and *HOXA11* promoters in DNA from 12 tumors (six each of the 3q8pq20 and non-3q8pq20 subtypes) revealed that only *HOXA9* was differentially methylated. Significant differences in methylation levels of *HOXA9* were observed amongst the 52 oral SCCs with respect to genomic subtype and nodal status (p = 0.014, and p = 0.024, respectively, Wilcoxon rank sum test). High levels of *HOXA9* methylation and low levels of expression in oral SCC cell lines were observed compared to HaCaT, a non-tumorigenic keratinocyte cell line. Re-expression of *HOXA9* in the SCC4 oral cancer cell line resulted in diminished proliferation and colony formation.

**Conclusions:**

*HOXA9* methylation is frequent in oral cancers and levels are higher in tumors with greater risk of metastasis. Expression of *HOXA9* is low in cells with high levels of methylation and reduced expression appears to confer a growth advantage.

## Background

Metastasis to the cervical (neck) lymph nodes is one of the most significant clinical factors responsible for death from oral squamous cell carcinoma (SCC). Currently, there are no satisfactory clinical, imaging, pathologic or molecular techniques that can reliably determine if neck metastases are present at the time of surgery to remove the primary tumor. Therefore, patients and physicians frequently elect to remove the cervical lymph nodes (neck dissection) at the time the tumor is excised if the chance of metastasis is > 20% based on current imperfect risk assessment capability. This surgical procedure is lengthy, complex, and risky with high morbidity due to functional deficits and disfigurement. Being able to determine which patients do not need such surgery would have a substantial, immediate clinical benefit.

We recently reported that two subtypes of oral SCC distinguished by tumor genomic aberrations differ in risk for metastasis [1]. One subtype, the 3q8pq20 subtype, is characterized by the presence of one or more of the recurrent copy number aberrations, +3q, -8p, +8q and/or +20 and has a substantial risk of metastasis (46%). The other subtype (non-3q8pq20) lacks these copy number alterations and is associated with a low risk of metastasis (7%). These initial studies, which were replicated in a small independent cohort, indicated that non-3q8pq20 status has 93% negative predictive value (NPV), *i.e*., ability to predict that these cases do not have neck metastases, and thus do not need neck dissection.

The non-3q8pq20 tumors lack chromosome level instability, which suggests that development of these tumors could be associated with other, copy number neutral, mechanisms, such as microsatellite instability or epigenetic alterations. Microsatellite instability is not common in oral SCC from western countries, whereas genome-wide alterations in methylation patterns are observed [2,3]. Analysis of a head and neck cancer patient cohort [2] for which both copy number and methylation measurements were available (NCBI GEO accession numbers GSE20939 and GSE20742, respectively) found 15 loci significantly differentially methylated in 3q8pq20 compared to non-3q8pq20 tumors and normal oral tissue [1]. To investigate whether these loci are a potential biomarker for distinguishing 3q8pq20 and non-3q8pq20 tumors, we investigated the methylation status of the loci in an oral SCC cohort in which 3q8pq20 status had been determined [1]. The overall goal was to develop a simple assay for 3q8pq20 status utilizing a panel of differentially methylated loci that could be performed on tissue samples obtained by a non-invasive technique prior to surgery and thus guide decisions regarding the need for neck dissection.

## Methods

### Patients and tissue samples

The study was approved by the Institutional Review Board of the University of California San Francisco (H7867-23910-05). Formalin fixed paraffin embedded (FFPE) SCC surgical resection specimens were available from 52 cases of the previously published SCC cohort#2 [1] and included oral cavity sites–tongue, gingiva, floor of mouth, retromolar trigone and buccal mucosa. Associated clinical data were obtained through the University of California San Francisco Oral Cancer Tissue Bank and Cancer Registry (Table 1 and Additional file 1: Table S1 of reference [1]). Patient consent was obtained for use of all specimens.

**Table 1:** Patient characteristics

**Sample**	**Site**	**Nodal status**	**3q8pq20**
AB003	Retromolar Region	N0	Yes
AB004	Gingiva	N0	Yes
AB007	Floor of Mouth	N+	Yes
AB010	Tongue	N0	Yes
AB011	Tongue	N0	Yes
AB014	Retromolar Region	N+	Yes
AB015	Tongue	N+	Yes
AB017	Buccal Mucosa	N+	Yes
AB018	Floor of Mouth	N+	Yes
AB019	Floor of Mouth, tongue	N+	Yes
AB020	Hard Palate	N+	Yes
AB021	Tongue	N0	no
AB023	Tongue	N0	Yes
AB025	Gingiva	N0	No
AB026	Retromolar Region	N0	Yes
AB029	Floor of Mouth, tongue, buccal mucosa	N0	Yes
AB031	Tongue	N0	Yes
AB032	Buccal Mucosa	N0	Yes
AB033	Retromolar Region	N+	Yes
AB034	Buccal Mucosa	N0	No
AB035	Tongue	N0	No
AB039	Gingiva	N0	Yes
AB041	Tongue	N0	Yes
AB042	Tongue	N0	Yes
AB045	Gingiva	N0	No
AB048	Tongue	N0	Yes
AB049	Tongue	N0	No
AB051	Tongue	N+	Yes
AB054	Buccal Mucosa	N+	Yes
AB055	Floor of Mouth	N0	Yes
AB056	Retromolar Region	N+	Yes
AB059	Tongue	N+	Yes
AB060	Tongue, Floor of Mouth	N+	Yes
AB061	Buccal Mucosa	N0	Yes
AB062	Gingiva	N0	No
AB063	Tongue	N0	Yes
AB064	Buccal Mucosa	N0	Yes
AB066	Tongue	N0	Yes
AB067	Floor of Mouth	N0	Yes
AB068	Gingiva	N0	No
AB070	Floor of Mouth	N0	Yes
AB071	Hard Palate	N0	Yes
AB073	Gingiva	N+	No
AB077	Floor of Mouth	N0	Yes
AB079	Tongue	N0	No
AB080	Tongue	N0	No
AB081	Gingiva	N+	Yes
AB082	Floor of Mouth	N+	Yes
AB083	Floor of Mouth, tongue, gingiva	N+	Yes
AB084	Gingiva	N+	Yes
AB085	Tongue	N0	Yes
AB086	Floor of Mouth	N0	No

### Tumor cell lines

Human oral tongue SCC cell lines SCC4, SCC9, SCC15 and SCC25 were obtained from the American Type Culture Collection (Manassas, VA), BICR16, H357, H103, PE/CA-PJ15, and PE/CA-PJ49 from the Health Protection Agency Culture Collections (HPA, Salisbury, UK), CAL33 from Deutsche Sammlung von Mikroorganismen und Zellculturen GmbH (DSMZ, Braunschweig, Germany), and OSC20 from the Japanese Collection of Research Biosources (Osaka, Japan). The DOK cell line, derived from a human oral dysplasia was obtained from the Health Protection Agency Culture Collections (HPA, Salisbury, UK) and HaCaT, a skin keratinocyte line was from the Deutsche Sammlung von Mikroorganismen und Zellculturen GmbH (DSMZ, Braunschweig, Germany). Cells were propagated according to the methods recommended by the suppliers.

### Bisulfite conversion and pyrosequencing

The DNA concentration was quantified by Quant-iT™ dsDNA BR Assay (Life Technologies, Grand Island, NY). A total of 200 ng of each DNA sample was bisulfite converted with the EZ DNA Methylation-Direct Kit (Zymo Research, Orange, CA). EpiTect Control DNA (QIAGEN, Germantown, MD) was used as methylated and unmethylated control DNA. PyroMark assays (QIAGEN, Germantown, MD) were used to determine methylation status of *HOXA9* (Hs_HOXA9_05_PM) and *MT1A* (HS_MT1A_02_PM). A custom assay was designed for *HOXA11* (Forward: biotin-5’-AGAGGTAGGTAGGGAAGATG-3’ , Reverse: 5’-CCCCTCCCATAAACTTACTCTAAA-3’ , Sequencing: 5’-ACACTCTCTCATTCATAATC-3’). Bisulfite PCR was performed using the PyroMark PCR kit (QIAGEN, Germantown, MD) and amplification was carried out by an initial incubation at 95°C for 15 min, followed by 45 cycles of 94°C for 30 sec, 55°C for 30 sec, 72°C for 30 sec. A final incubation was carried out at 72°C for 10 min. The biotinylated PCR product was purified and subjected to pyrosequencing using the PyroMark Q24 System and PyroMark Q24 gold reagents (QIAGEN, Germantown, MD). Data were analyzed by PyroMark Q24 2.0.6 software. The HOXA9 assay (Hs_HOXA9_05_PM) includes three CpG islands. Methylation level was assigned as the mean of the three sites.

### RT-PCR

Total RNA was extracted from cell lines using TRIzol® Reagent (Life Technologies, Grand Island, NY) according to the manufacturer’s instructions. RNA quantity was determined with a NanoDrop 2000 Spectrophotometer (Thermo Fisher Scientific Inc., Asheville NC, US) and RNA integrity was assessed with the Bioanalyzer™ (Agilent Technologies, Inc., average RIN for cell lines = 8.2). A fixed amount of total RNA (500 ng) per each sample was reverse transcribed with iScript™ Select cDNA Synthesis kit (Bio-Rad, Hercules CA, US).

Standard TaqMan qRT-PCR Gene Expression assays were conducted in triplicate to quantify *HOXA9* expression levels relative to *GUSB*. Duplex PCR was performed with the FAM labeled Taqman assay for *HOXA9* (Hs00365956_m1) and VIC labeled Taqman assay for *GUSB* (Hs00939627_m1). Reactions (10 μL per well) included 5 μL TaqMan Gene Expression Master Mix (Life Technologies, Grand Island, NY), 0.5 μL 20× Gene Expression Assay Mix, and 1 μL cDNA diluted to a final concentration of 10 ng/μL. Assay plates were run on an Applied Biosystems 7900HT detection system using standard settings (cycling program included 2 min incubation at 50°C and 10 min incubation at 95°C followed by 40 cycles of 95°C for 15 sec and 60°C for 1 min). Data values (Cycle Threshold [Ct] values) were extracted from each assay with the SDS v2.0 software tool (Life Technologies, Grand Island, NY). Gene expression values were derived from the equation: ΔCt = (Ctgene–CtGUSB) and expressed as 2^-ΔCt^.

For end point PCR, the primer sequences used to amplify *GUSB* were 5’-TGCGCACAAGAGTGGTGCTGA-3′ and 5′-TCGACCCCATTCACCCACACGA-3′. The primers for *BRCA1* have been reported previously [4]. Amplification reactions used the HotStarTaq Master Mix kit (QIAGEN, Germantown, MD). An initial incubation at 95°C for 15 min was followed by 45 cycles of 94°C for 30 sec, 55°C (*BRCA1*) or 60°C (*GUSB*) for 30 sec, 72°C for 30 sec and a final incubation at 72°C for 10 min. Nucleotide free water was used for the negative control and Universal Human Reference RNA (Agilent technologies, Santa Clara, CA) was used for the positive control.

### Establishment of the SCC4 cell line with the HOXA9 inducible-expression construct

To generate stable, inducible cell lines expressing *HOXA9* upon doxycycline induction, *HOXA9* cDNA (BC10023, Open Biosystems) was subcloned into pLVX-tight-puro (Clontech, CA). SCC4 cells were transfected with pLVX-Tet-On and pLVX-HOXA9 using Lipofectamine LTX (Life Technologies, Grand Island, NY)) according to the manufacturer’s instructions. Three days after transfection, stable clones were selected by culturing for 2 weeks in medium supplemented with 1 μg/mL Puromycin (Sigma-Aldrich) and 300 μg/mL G418 (Roche). These SCC4-HOXA9 cells were cultured with 1 μg/mL doxycycline to induce *HOXA9* expression, which was verified by qPCR and western blotting. Empty vector control SCC4 cells (SCC4-empty vector) were generated by transfecting with pLVX-Tet-On and pLVX-tight-puro and selection by culturing for 2 weeks in medium supplemented with 1 μg/mL puromycin and 300 μg/mL G418.

### Proliferation and colony formation assays

To assess cell proliferation, 500 SCC4-HOXA9 and SCC4-empty vector cells were seeded in 96-well culture plates. After allowing cells to attach and grow for 24 hours, 100 μL of culture medium supplemented with 1 μg/mL doxycycline was added to the cultures. Plates containing six replicate wells of each cell type were harvested over a period of nine days and proliferation was measured using the CyQUANT® NF assay (Life Technologies, Grand Island, NY).

Colony formation assays were performed by seeding cells in six-well plates at a density of 1000 cells/well in 2 mL complete medium. After culturing for two weeks, colonies were stained with 0.5% crystal violet in 2% ethanol and colonies were counted.

### Statistical analysis

A Wilcoxon rank sum test was used to assess differential methylation between groups. A T-test was used to assess differences in proliferation and colony formation assays.

## Results

### Pilot study evaluating methylation status of *HOXA9*, *HOXA11* and *MT1A*

**Figure 1:**
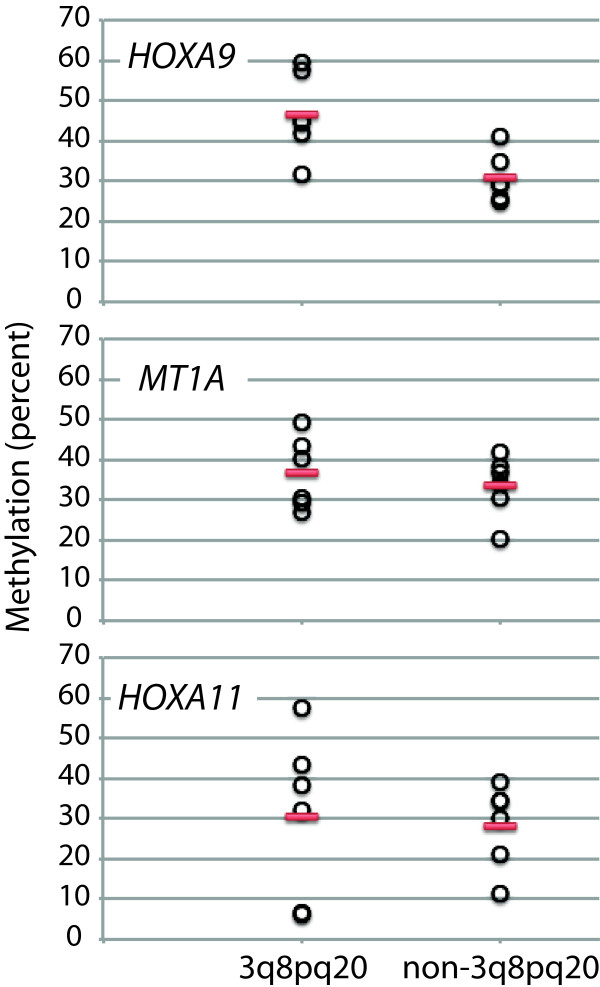
**Pilot study evaluating methylation of *HOXA9*, *MT1A *and *HOXA11 *in 3q8pq20 and non-3q8pq20 tumors.** Methylation levels of the gene promoters are shown for individual 3q8pq20 and non-3q8pq20 tumors (open circles). The mean level is indicated by the red bar. Differential methylation in the two oral cancer subtypes is evident for *HOXA9*.

Methylation and tumor genome copy number data reported by Poage and colleagues [2] were accessioned and cases assigned to the 3q8pq20 or non-3q8pq20 subtypes as described previously [1]. Information on nodal status was not available for these cases. Methylation levels were compared between tumor subtypes, and this analysis revealed 15 loci that were differentially methylated between 3q8pq20 and non-3q8pq20 plus normal tissue (Additional file 2: Figure S1, Additional file 1: Table S1). *HOXA9*, *HOXA11* and *MT1A* were selected for further study, because promoters of these genes were consistently highly methylated in 3q8pq20 cases. Methylation status of the CpG islands in these promoters was evaluated by pyrosequencing in 12 tumors (six 3q8pq20 and six non-3q8pq20 subtypes) from the previously reported SCC cohort#2 for which copy number and clinical follow up data were available [1]. In this pilot study, only *HOXA9* appeared to be differentially methylated between the two subtypes (Figure 1).

### Association of *HOXA9* methylation with risk for metastasis

**Figure 2:**
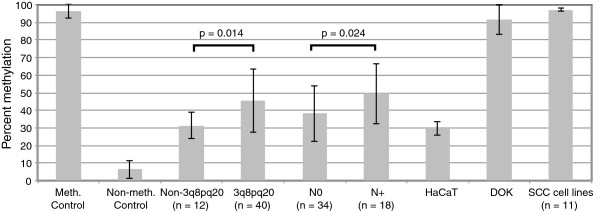
**Methylation of *HOXA9 *** **in oral cancers and cell lines. Shown are the mean and standard deviation.**

**Figure 3:**
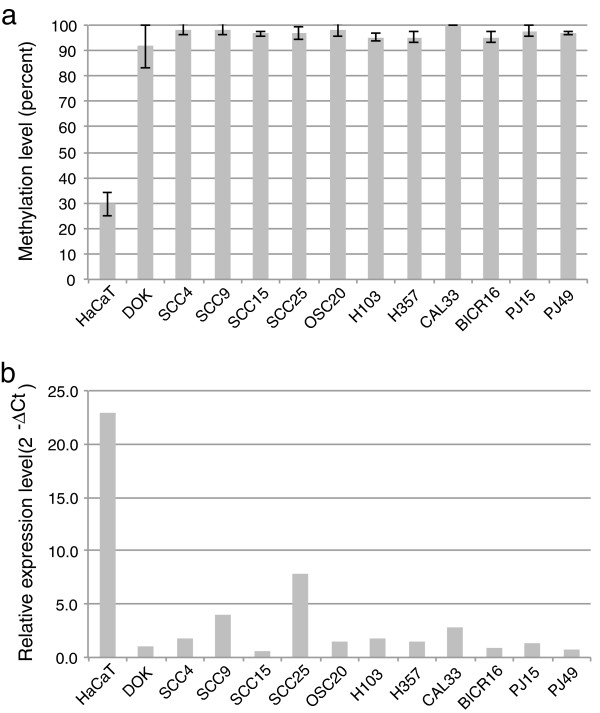
**HOXA9 is methylated and expressed at low levels in oral cancer cell lines. a**. Methylation levels (mean and standard deviation) as determined by pyrosequencing. **b**. Transcript levels determined by quantitative RT-PCR

The methylation status of *HOXA9* was further investigated in cell lines and 40 additional tumors from SCC cohort#2 [1] by pyrosequencing (Figure 2, Additional file 1: Table S2). The *HOXA9* promoter is nearly completely methylated in oral cancer cell lines and to a slightly lesser extent in DOX, a cell line derived from dysplastic tissue (Figure 3a). By contrast, only 30% methylation of *HOXA9* was observed in the non-tumorigenic HaCaT cells. The mean level of methylation of non-3q8pq20 tumors (31%) was similar to HaCaT cells and significantly less than that of 3q8pq20 tumors (45%) or clinically normal epithelium from four cancer patients (16%). We also observed a significant difference in methylation when comparing node positive (49%) and node negative cases (38%), irrespective of genomic subtype.

### Re-expression of *HOXA9* inhibits growth

In oral cancer cell lines with nearly complete methylation of the *HOXA9* promoter, *HOXA9* expression levels are low (Figure 3). By contrast, *HOXA9* expression levels are higher in HaCaT cells with only partial methylation of the promoter (Figure 3). These observations suggest that promoter methylation is frequently a mechanism whereby expression of *HOXA9* expression can be repressed in oral cancer and pre-cancer cells.

**Figure 4:**
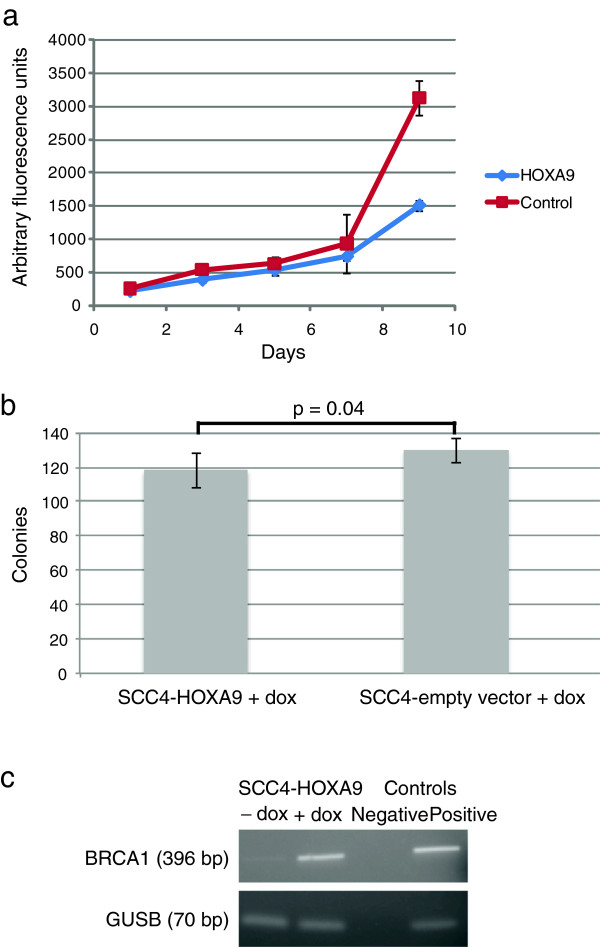
**Re-expression of *HOXA9 *represses proliferation and colony formation and induces expression of *BRCA1*. a**. Proliferation of SCC4-HOXA9 and SCC4-empty vector cells. **b**. Colony formation by SCC4-HOXA9 and SCC4-empty vector cells. **c**. Endpoint PCR analysis of *BRCA1* expression.

To investigate the possible functional significance of reduced expression of *HOXA9*, we re-expressed *HOXA9* in SCC4 tongue cancer cells (SCC4-HOXA9), which resulted in diminished proliferation compared to control SCC4 cells with an empty vector construct (SCC4-empty vector, Figure 4a). We also observed that fewer (p = 0.04), and smaller colonies were formed by SCC4-HOXA9 cells in colony formation assays compared to SCC4 cells with an empty vector construct (Figure 4b and Additional file 2: Figure S2). In breast cancer cells, *HOXA9* has been shown to directly regulate *BRCA1* expression and to suppress growth and survival [4]. Expression of *HOXA9* in SCC4 cells also resulted in increased expression of *BRCA1* (Figure 4c), suggesting that, in addition to promoting growth, reduced expression of *HOXA9* may contribute to tumor genome instability.

## Discussion

The risk of metastasis in oral cancer patients is associated with the status of copy number alterations at chromosome 3q, 8p, 8q and 20. The presence of any one of these aberrations (3q8pq20 genomic subtype) is associated with a substantial risk of metastasis (46%), while absence of alterations in all of these regions (non-3q8pq20 subtype) shows greater promise as a biomarker for low risk of metastasis (93% NPV). If validated in further larger clinical studies, determination of non-3q8pq20 status prior to surgery could identify those patients at low risk of metastasis who could be spared the extra surgery of an elective neck dissection. While non-3q8pq20 status could be determined prior to surgery by profiling tumor biopsies for copy number alterations, assignment of non-3q8pq20 status might be ambiguous if no copy number changes were present on other chromosome arms. Therefore, we investigated whether differential methylation of loci could act as a surrogate and identify 3q8pq20 and non-3q8pq20 subtypes. Two of the candidate loci (*HOXA11* and *MT1A*), selected from analysis of published data, failed to validate in our cohort. Only *HOXA9* was found to be differentially methylated in 3q8pq20 compared to non-3q8pq20 tumors, as well as between node positive and node negative cases. Although the differences in methylation level reached statistical significance, they are modest and would probably not result in a robust clinical test for nodal status.

Homeobox (HOX) genes are transcription factors with roles in development, regulating patterning during embryogenesis and maintaining differentiated states. De-regulated expression of HOX genes is reported in cancers [5]. They can be overexpressed and act as oncogenes or they can act as tumor suppressors with expression down regulated via promoter methylation. Our data are consistent with *HOXA9* acting as a tumor suppressor in oral cancer. Methylation of *HOXA9* has been reported previously in oral cavity cancer [6], and methylation and loss of expression of *HOXA9* reported in breast [4,7,8], lung [9], ovarian [10] and bladder cancer [11], whereas *HOXA9* is well known to act as an oncogene in leukemia [5]. The tumor suppressor function of *HOXA9* has been extensively investigated in breast cancer where it has been shown that *HOXA9* directly regulates *BRCA1*[4] and a number of other genes involved in invasion, growth and metastasis [7]. While we show here that *HOXA9* also appears to positively regulate *BRCA1* expression in oral cancer cells, further studies will be required to fully understand how *HOXA9* functions as a tumor suppressor in oral cancer. Indeed, the oncogenic and tumor suppressive activities of deregulated *HOXA9* expression in different tissues highlight the importance of tissue context for the functioning of deregulated developmental genes in cancer.

## Conclusions

The *HOXA9* promoter is frequently methylated in oral SCC and diminished expression of *HOXA9* confers a growth advantage to oral SCC cells. Higher levels of *HOXA9* methylation are present in tumors with greater risk of metastasis.

## Abbreviations

SCC: Squamous cell carcinoma; NPV: Negative predictive value.

## Competing interests

The authors declare that they have no competing interests.

## Authors’ contributions

KU, RV and BH carried out the molecular assays and cell culture experiments. AB, BLS and DGA assembled the patient cohort and associated clinical and molecular information. KU and DGA drafted the manuscript. DGA conceived of the study, and participated in its design and coordination. All authors read and approved the final manuscript.

## Pre-publication history

The pre-publication history for this paper can be accessed here:

http://www.biomedcentral.com/1471-2407/14/353/prepub

## Supplementary Material

Additional file 1: Table S1 Comparison of methylation levels in 3q8pq20 cancers vs. non-3q8pq20 cancers + normals (data from NCBI GEO, GSE20939 and GSE20742). **Table S2.** Methylation status of *HOXA9* in oral cancers. Click here for file

Additional file 2: Figure S1 Differentially methylated loci. Copy number and methylation data from Poage *et al*. [1] were obtained from NCBI GEO (Accession numbers GSE20742 and GSE20939, respectively). Copy number data were segmented and samples were assigned to the 3q8pq20 or non-3q8pq20 subtype based on the presence of copy number changes, +3q, -8p, +8q, and/or +20 as described previously [2]. A nonlinear transformation was applied to the methylation data beta values [s = sqrt(beta) - sqrt(1 - beta)], which increases the Gaussian character of the data and has the effect of reducing the number of false positives. The transformed data were then quantile normalized across samples. Probes were tested for differential methylation between 3q8pq20 and non-3q8pq20 subtypes plus normal cases using the limma package. The probes for each comparison were filtered on absolute mean difference in methylation level (> 0.05) and adjusted p-value (< 0.05, FDR) [3]. This analysis yielded 15 probes differentially methylated between 3q8pq20 and non-3q8pq20 samples. **Figure S2.** Colony formation by SCC4 cells expressing *HOXA9* and control SCC4 cells. Shown are images of six-well plates following staining with crystal violet. SCC4-HOXA9 and SCC4-empty vector cells were cultured in the presence of 1 μg/mL doxycycline. Smaller colonies are present in the plates of SCC4-HOXA9 cells in which *HOXA9* was re-expressed (top two panels) compared to SCC4 cells with an empty vector (SCC4-empty vector, bottom two panels). Click here for file
