# Supplementary material for: Investigation of HOXA9 promoter methylation as a biomarker to distinguish oral cancer patients at low risk of neck metastasis
Source: BMC Cancer. 2014 May 21;14:353. doi: 10.1186/1471-2407-14-353 (PMC4045880; doi:10.1186/1471-2407-14-353)
Supplement: Additional file 2: Figure S1 — Differentially methylated loci. Copy number and methylation data from Poage et al. [1] were obtained from NCBI GEO (Accession numbers GSE20742 and GSE20939, respectively). Copy number data were segmented and samples were assigned to the 3q8pq20 or non-3q8pq20 subtype based on the presence of copy number changes, +3q, -8p, +8q, and/or +20 as described previously [2]. A nonlinear transformation was applied to the methylation data beta values [s = sqrt(beta) - sqrt(1 - beta)], which increases the Gaussian character of the data and has the effect of reducing the number of false positives. The transformed data were then quantile normalized across samples. Probes were tested for differential methylation between 3q8pq20 and non-3q8pq20 subtypes plus normal cases using the limma package. The probes for each comparison were filtered on absolute mean difference in methylation level (> 0.05) and adjusted p-value (< 0.05, FDR) [3]. This analysis yielded 15 probes differentially methylated between 3q8pq20 and non-3q8pq20 samples. Figure S2. Colony formation by SCC4 cells expressing HOXA9 and control SCC4 cells. Shown are images of six-well plates following staining with crystal violet. SCC4-HOXA9 and SCC4-empty vector cells were cultured in the presence of 1 μg/mL doxycycline. Smaller colonies are present in the plates of SCC4-HOXA9 cells in which HOXA9 was re-expressed (top two panels) compared to SCC4 cells with an empty vector (SCC4-empty vector, bottom two panels). [file 1471-2407-14-353-S2.pdf]

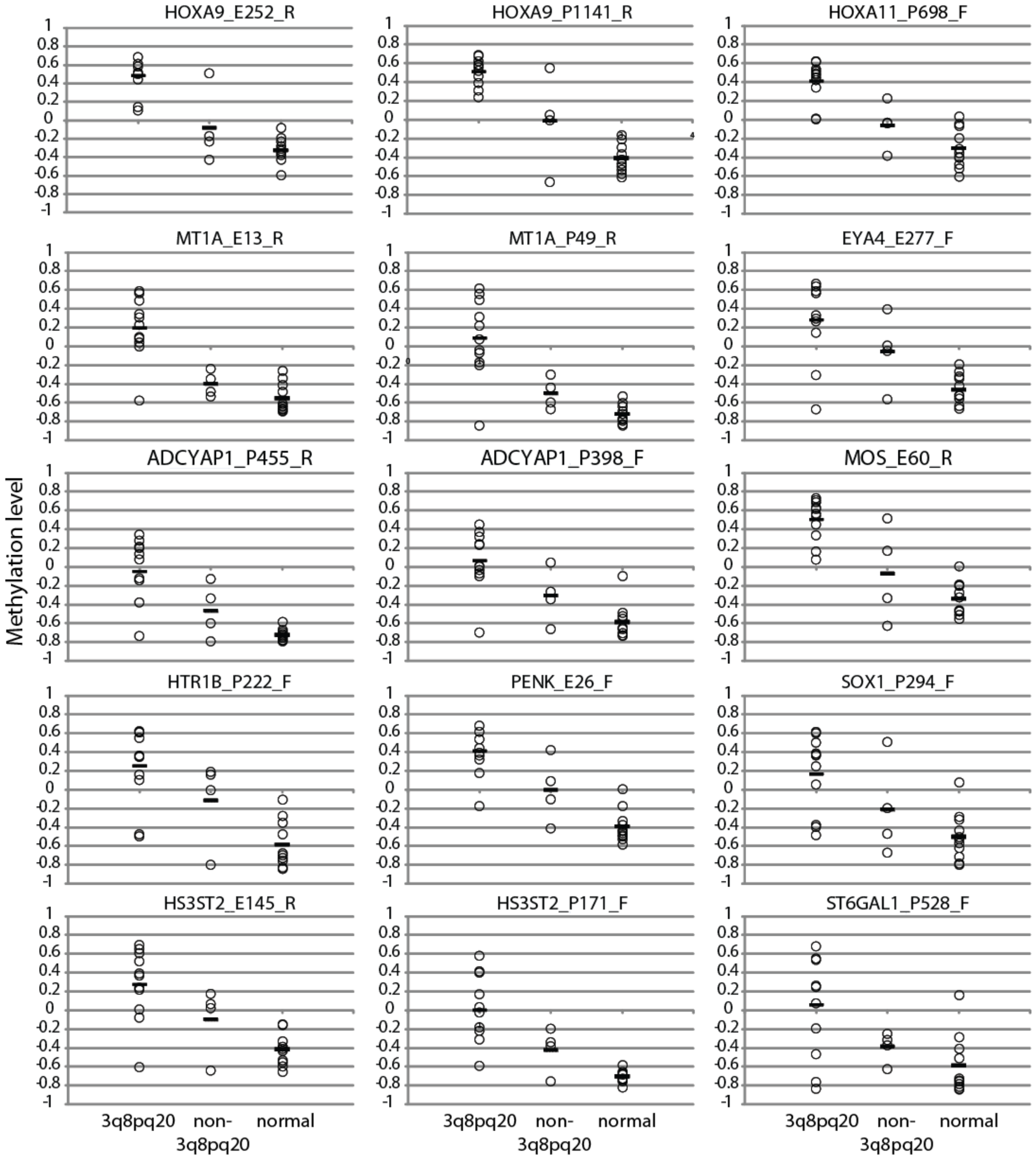

Figure S1. Differentially methylated loci. Copy number and methylation data from Poage *et al*. [1] were obtained from NCBI GEO (Accession numbers GSE20742 and GSE20939, respectively). Copy number data were segmented and samples were assigned to the 3q8pq20 or non-3q8pq20 subtype based on the presence of copy number changes, +3q, -8p, +8q, and/or +20 as described previously [2]. A nonlinear transformation was applied to the methylation data beta values [s = sqrt(beta) - sqrt(1 - beta)], which increases the Gaussian character of the data and has the effect of reducing the number of false positives. The transformed data were then quantile normalized across samples. Probes were tested for differential methylation between 3q8pq20 and non-3q8pq20 subtypes plus normal cases using the limma package. The probes for each comparison were filtered on absolute mean difference in methylation level (> 0.05) and adjusted p-value (< 0.05, FDR) [3]. This analysis yielded 15 probes differentially methylated between 3q8pq20 and non-3q8pq20 samples.

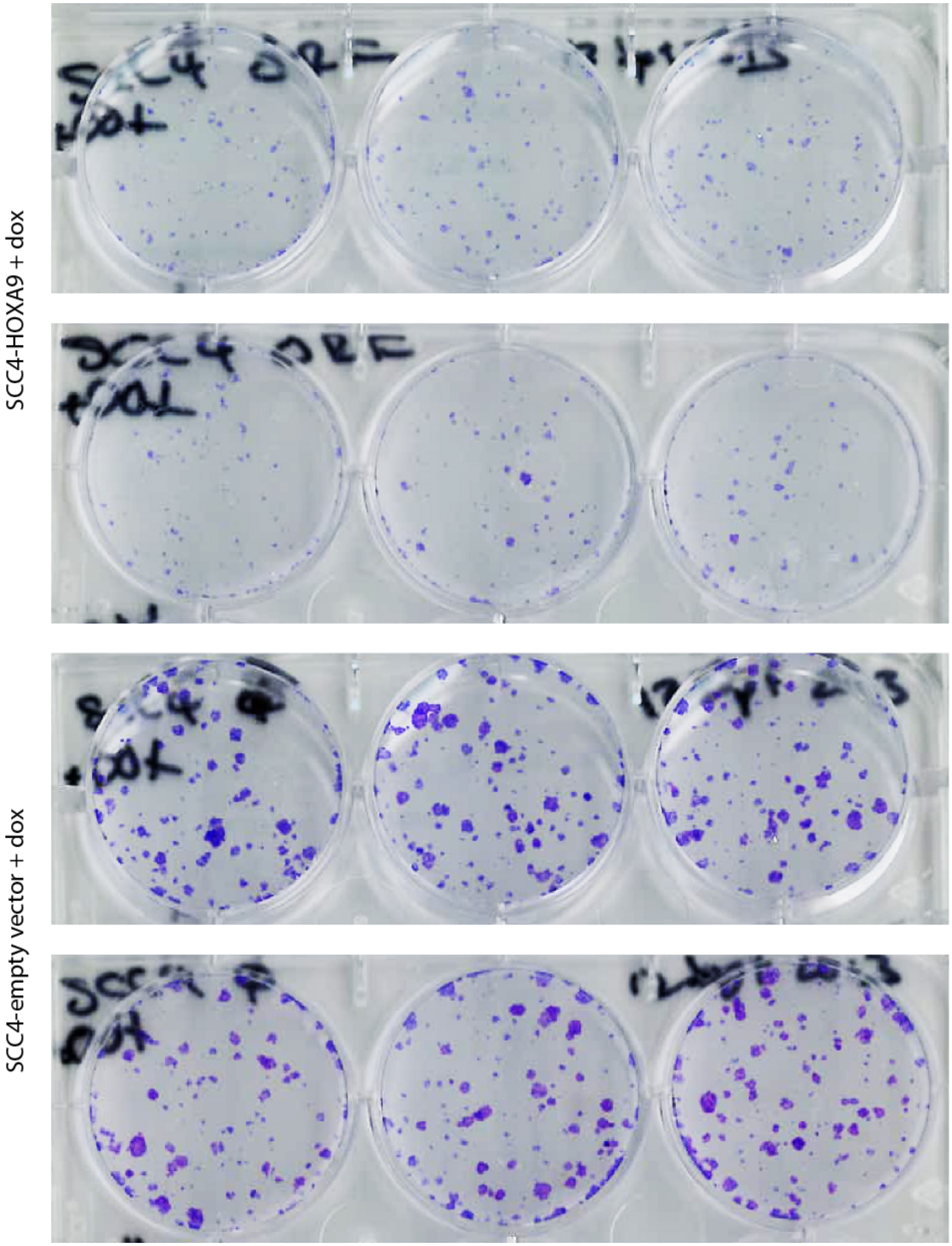

Figure S2. Colony formation by SCC4 cells expressing *HOXA9* and control SCC4 cells. Shown are images of six-well plates following staining with crystal violet. SCC4-HOXA9 and SCC4-empty vector cells were cultured in the presence of 1 µg/mL doxycycline. Smaller colonies are present in the plates of SCC4-HOXA9 cells in which *HOXA9* was re-expressed (top two panels) compared to SCC4 cells with an empty vector (SCC4-empty vector, bottom two panels).

References

1. Poage GM, Christensen BC, Houseman EA, McClean MD, Wiencke JK, Posner MR, Clark JR, Nelson HH, Marsit CJ, Kelsey KT: **Genetic and epigenetic somatic alterations in head and neck squamous cell carcinomas are globally coordinated but not locally targeted**. *PloS one* 2010, **5**(3):e9651.
2. Bhattacharya A, Roy R, Snijders AM, Hamilton G, Paquette J, Tokuyasu T, Bengtsson H, Jordan RC, Olshen AB, Pinkel D *et al*: **Two distinct routes to oral cancer differing in genome instability and risk for cervical node metastasis**. *Clinical cancer research : an official journal of the American Association for Cancer Research* 2011, **17**(22):7024-7034.
3. Benjamini Y, Hochberg Y: **Controlling the false discovery rate: a practical and powerful approach to multiple testing**. *Journal of the Royal Statistical Society Series B* 1995, **57**:289-300.
